# Precipitation of complex antibody solutions: influence of contaminant composition and cell culture medium on the precipitation behavior

**DOI:** 10.1007/s00449-019-02103-y

**Published:** 2019-03-18

**Authors:** Steffen Großhans, Susanna Suhm, Jürgen Hubbuch

**Affiliations:** Institute of Process Engineering in Life Sciences, Section IV: Biomolecular Separation Engineering, Karlsruhe Institute of Technology (KIT), 76131 Karlsruhe, Germany

**Keywords:** Preparative protein precipitation, Polyethylene glycol, Industrial monoclonal antibody, Contaminants, Cell culture medium

## Abstract

Preparative protein precipitation is known as a cost-efficient and easy-to-use alternative to chromatographic purification steps. This said, at the moment, there is no process for monoclonal antibodies (mAb) on the market, although especially polyethylene glycol-induced precipitation has shown great potential. One reason might be the highly complex behavior of each component of a crude feedstock during the precipitation process. For different investigated mAbs, significant variations in the host cell protein (HCP) reduction are observed. In contrast to the precipitation behavior of single components, the interactions and interplay in a complex feedstock are not fully understood yet. This work discusses the influence of contaminants on the precipitation behavior of two different mAbs, an IgG1, and an IgG2. By spiking the mAbs with mock solution, a complex feedstock could successfully be mimicked. Spiking contaminants influenced the yield and purity of the mAbs after the precipitation step, compared to the precipitation behavior of the single components. The mixture showed a decrease in the contaminant and mAb solubility. By re-buffering the mock solution prior to spiking, special salts, small molecules like amino acids, vitamins, or sugars could be depleted while larger ones like HCP or DNA were still present. Therefore, it was possible to distinguish the influence of small molecules and larger ones. Hence, mAb–macromolecular interaction could be identified as a possible reason for the observed higher precipitation propensity, while small molecules of the cell culture medium were identified as solubilisation factors during the precipitation process.

## Introduction

Since the development of the first recombinant biopharmaceuticals, like insulin or monoclonal antibodies (mAb) in the 1980s, the industry has been growing rapidly [1]. The relative low potency of mAbs in combination with the treatment of chronic diseases and cancer currently leads to a high amount for each dose and, consequently, a considerable amount of total mAbs produced. Their success made mAbs one of the most expensive drug classes on the market [2]. In the past, the focus was set on bringing innovative products fast to the market and less on developing cost-efficient processes. Additionally, there was hardly a link between manufacturing costs and the cost of the final product [3]. Restricted healthcare budgets and the development of bio-similars, the first monoclonal antibody having been approved in 2013 by the European Medicines Agency (EMA), however, raised the need for a more economic production [4, 5].

Currently, mAbs are mostly manufactured using platform processes, consisting of batch/fed-batch or perfusion cell cultures, followed by the respective downstream process. The downstream process is mainly a combination of chromatography steps. For the capture step, Protein A affinity chromatography is still the most common technique. High resin costs and slow volumetric throughput make this step an easy-to-use, but expensive unit operation [6]. In upstream process development (USP), higher cell densities and cell culture titers led to an increase in process efficiency [7]. With perfusion cell cultures, titers up to 25 mg/mL were reported [8]. These efforts lead to a demand for new and cost-efficient alternative techniques for downstream process development (DSP). These methodologies need to be comparable to the purification performance of the chromatography step.

Selective protein precipitation has shown promising results such as cost-efficient alternatives. Particularly for small-scale production, PEG as precipitation agent has shown its potential as easily scalable and simple technique [9, 10]. At best, the mAb is selectively precipitated while the contaminants stay in solution. New techniques such as high-throughput process development (HTPD), design of experiment (DoE), or model-based approaches can help to make precipitation process development faster and with less brute force [11–13]. However, huge variations in the host cell protein (HCP) reduction were seen for different investigated mAbs [14]. Especially for the development of a generic mAb platform process, a better understanding of the precipitation mechanism is mandatory. Most studies, hence, have aimed more at efficient process development of one mAb feedstock and less at a detailed understanding of the complex behavior of components among each other.

For single protein solutions, much effort was put into the understanding of the mechanism of PEG-induced protein precipitation [15–17]. In general, precipitation using PEG can be described with two theories. According to the theory of excluded volume, the polymers are reported to trap the solvent and sterically exclude proteins from the solvent region occupied by the polymer [18–20]. Because this is a steric effect, the selectivity of PEG-induced protein precipitation is given by the size of the proteins. Proteins with a larger hydrodynamic radius tend to precipitate at lower PEG concentrations compared to proteins with a smaller hydrodynamic radius. The theory of attractive depletion further introduces osmotic pressure as additional force to the precipitation process [21, 22]. This force is caused by the steric exclusion of the PEG molecules from the ambiance of the proteins, the depletion zone. When two depletion zones overlap, a PEG-free area is created, and a concentration gradient towards the bulk is formed. This gradient leads to the described osmotic pressure. In both theories, there is no direct interaction between the protein and the polymers described. All other forces between particles, such as electrostatic or hydrophobic interactions, are still valid. This fact makes PEG-induced protein precipitation a function of changes in process conditions, such as pH or conductivity.

In opposite to single protein solutions, there is only little research on the precipitation behavior of protein mixtures. Mahadevan and Hall developed a theory describing the precipitation of protein mixtures by nonionic polymers using statistical mechanics [23]. Coen and co-workers investigated the phase behavior of aqueous binary protein mixtures containing either lysozyme–chymotrypsinogen or lysozyme–ovalbumin [24]. Solms et al. developed a thermodynamic framework for the description of a phase diagram of a lysozyme–ovalbumin system [25]. Sieberz et al. investigated the influence of model proteins as contaminants on the precipitation behavior of mAbs during an polyelectrolyte preciptiation process [26]. All these findings suggest, on the one hand, that the protein–protein interaction influences the protein phase behavior and, on the other, that this influence is not simply a linear or otherwise geometric combination of pure species interaction terms [27].

When developing a preparative precipitation process, not only a binary protein system has to be looked at, but the challenge is rather to purify one protein out of a complex multi-component system. Beside other proteins such as host cell proteins (HCP), small molecules like endotoxins, vitamins, or small cell culture media additives are present as contaminants as well and have to be removed [28]. The influence of small molecules on single proteins, known as osmolytes or co-solutes, is reported in many studies and can be explained by the preferential interaction theory [29–31]. According to the theory, originally developed by Timasheff et al., the co-solvents either preferentially interact with the protein or with water [32]. A preferential interaction of the co-solvent with the protein, also known as salting-in, leads to an increase in the colloidal stability of the protein. On the contrary, a preferential interaction with water leads to a destabilization of the system, known as salting-out.

Thus, the purification of a mAb out of a complex feed stock might be influenced by a multitude of contaminants and interactions. Changes in USP can lead to variations in pH, conductivity, contaminant profile, or product titer. These variations, in turn, can possibly influence the precipitation efficiency in terms of yield or purity. Especially with a focus on a platform-based approach to processes using precipitation, the focus should not be limited to the optimizing of one unit operation alone. An integrated process development of up- and downstream is necessary [33].

**Fig. 1:**
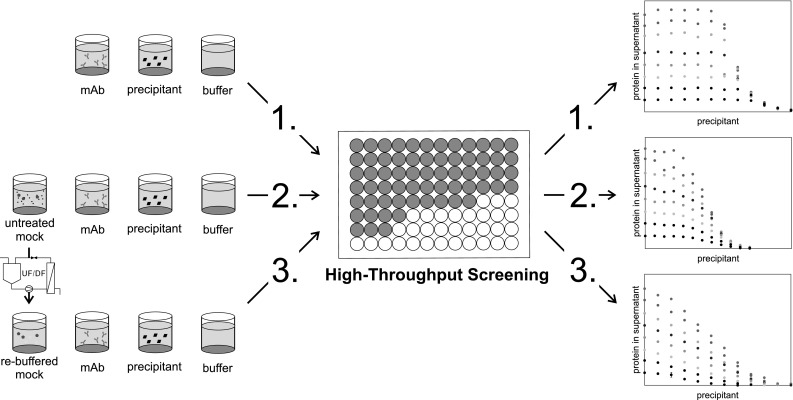
Schematic overview of the workflow for the precipitation experiments carried out in this study. For high-throughput experiments, mAb, mock, precipitant, and buffer stock solutions were mixed on a liquid-handling station with varying concentrations. Two different mock solutions were investigated. One was used without pretreatment, the other one was re-buffered prior to use. After phase separation, the supernatant of each system was investigated for mAb and contaminant concentration

**Table 1:** Characteristics of the utilized protein and contaminant stock solution

Molecule	mAbA purified	mAbA HCCF	mAbB purified	Mock solution untreated	Mock solution re-buffered
Type	IgG2	IgG2	IgG1	–	–
Molecular mass (kDA)	144.7	144.7	144.2	–	–
pI	8.3–8.5	8.3–8.5	8.3	–	–
mAb (mg/mL)	27.3	3.3	22.3	–	–
HCP (mg/mL)	$<0.01$	1.26	$<0.01$	1.04	3.18

The aim of this study is to investigate the influence of contaminants and their composition on the precipitation behavior on a preparative mAb precipitation. A workflow of the presented study is illustrated in Fig. 1. Precipitation curves of two purified mAbs at varying concentrations were generated using high-throughput experiments. These results were compared with the precipitation behavior of the same mAbs spiked with mock solution from a Chinese hamster ovary (CHO) cell line. Through this experimental setup, a complex feedstock was imitated. This allows to investigate the influence of a complex feedstock on different mAbs as well as different mAb concentrations. To explain the observed differences in the precipitation behavior, the mAbs were also spiked with purified re-buffered mock solution. Thereby, it was possible to exclude the variations caused by different pH values or conductivities and focus on the influence of protein–contaminant interactions. Finally, the re-buffered mock solution was spiked with fresh cell culture media prior to the precipitation to investigate the influence of the small molecules solved in the cell culture media on the observed interactions.

## Materials and methods

All precipitation experiments were carried out in 350 $\upmu$L polypropylene flat-bottom 96-well micro plates (Greiner Bio-One, Kremsmünster, Austria). For spectroscopic measurements, samples were diluted into Greiner UV-Star$^{\textregistered }$ micro plates (Greiner Bio-One, Kremsmünster, Austria).

**Fig. 2:**
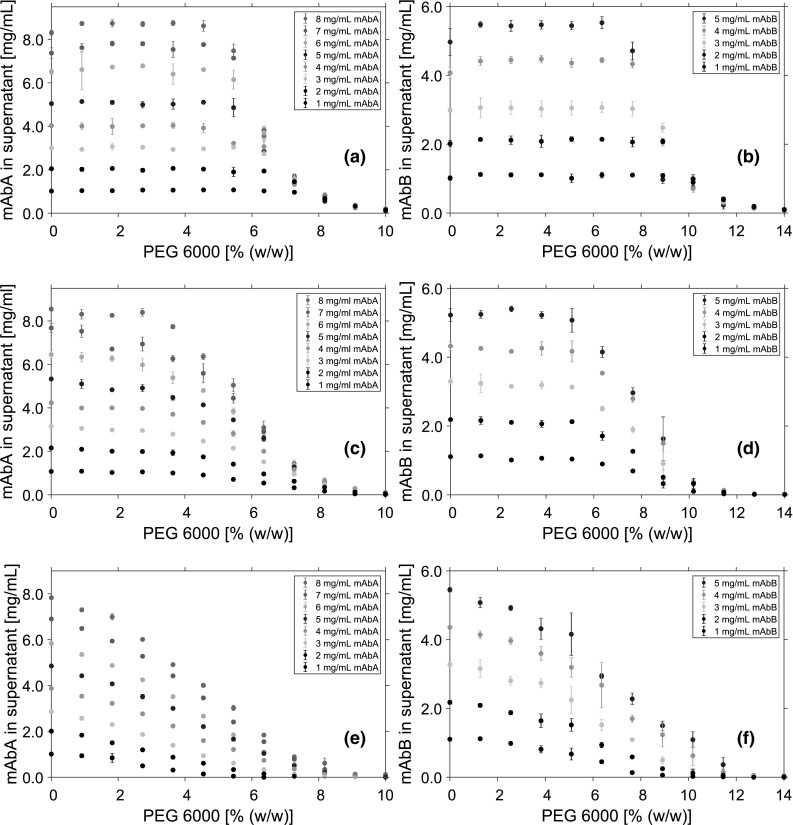
Solubility data of mAbA (**a, c, e**) and mAbB (**b, d, f**) in the supernatant after phase separation at varying mAb and PEG concentrations. Data points represent the mean value of triplicates. All experiments were conducted using 50 mM tris buffer at pH 7.5. **a**, **b** Purified mAb was precipitated. **c**, **d** The mAb was spiked with untreated mock solution containing 0.5 mg/mL HCP each. **e**, **f** The mAb was spiked with re-buffered mock solution containing 0.5 mg/mL HCP each. The mock solution was re-buffered to 50 mM tris pH 7.5

### Chemicals and stock solutions

All buffer solutions were prepared using water purified by a PURELAB Ultra water purification system (ELGA Labwater, High Wycombe, UK). As buffer substances, tris(hydroxymethyl)-aminomethane (Merck KGaA, Darmstadt, Germany) and tris hydrochloride (PanReac AppliChem, Darmstadt, Germany) were used. The polyethylene glycol (PEG) with a median molecular mass of 6000 g/mol was obtained from Merck KGaA (Darmstadt, Germany). All buffers were prepared with a buffer capacity of 50 mM. The desired pH was achieved by varying the amount of acid and basic component for each buffer. For the 40% (w/w) PEG stock solution, the buffer components were first dissolved in ddH$_{2}$O. Then, the appropriate amount of PEG was added. RPMI Medium 1640 (1 $\times$) + GlutaMAX™ (Thermo Fisher Scientific, Waltham, MA, USA) was used for spiking the samples with cell culture media.

**Fig. 3:**
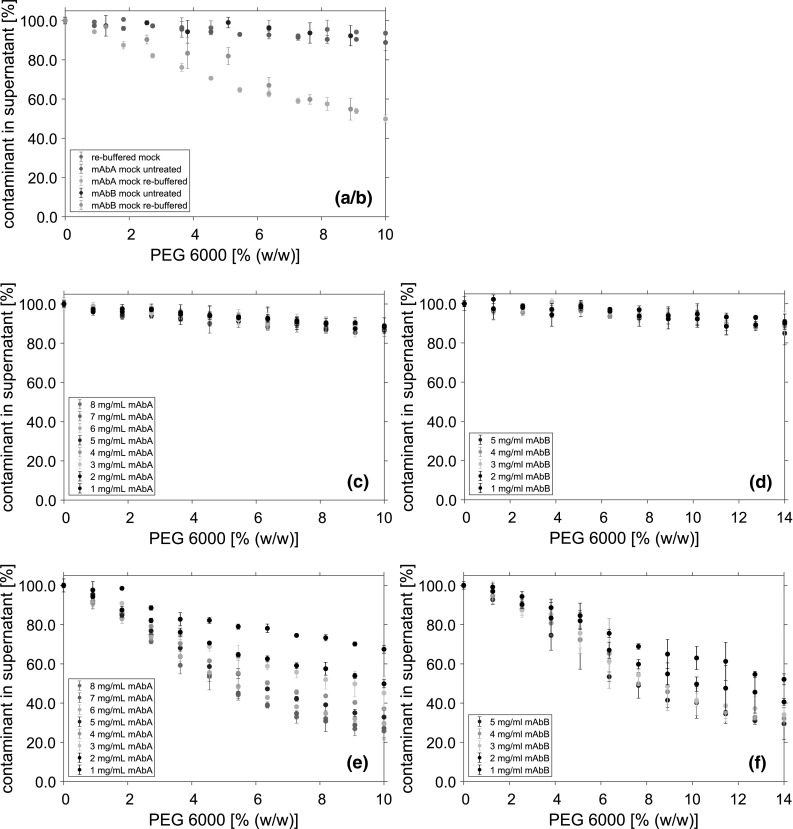
Solubility data of mAbA (**a, c, e**) and mAbB (**b, d, f**) in the supernatant after phase separation at varying mAb and PEG concentrations. Data points represent the mean value of triplicates. All experiments were conducted using 50 mM tris buffer at pH 7.5. **a**, **b** Purified mAb was precipitated. **c**, **d** The mAb was spiked with untreated mock solution containing 0.5 mg/mL HCP each. **e**, **f** The mAb was spiked with re-buffered mock solution containing 0.5 mg/mL HCP each. The mock solution was re-buffered to 50 mM tris pH 7.5

### Preparation of protein stock solutions

Two mAbs, A and B, were provided as purified solutions from Novartis (Basel, Switzerland). Additionally, mAbA was provided as harvested cell culture fluid (HCCF). The characteristics of the mAbs are listed in Table 1. Further more, Novartis supplied a mock solution originating from the same cell line used for the mAb fermentation. When mimicking a complex mAb solution, the mock solution was spiked with varying concentrations of purified mAbs. All protein solutions were filtered using $0.2\,\upmu$m cellulose acetate syringe filters (Sartorius AG, Göttingen, Germany) before usage or further pretreatment. All provided protein and feed solutions were stored at $-\,80\,^\circ$C for long-term and at $-\,30^\circ$C for short-term storage, after aliquotation.

**Fig. 4:**
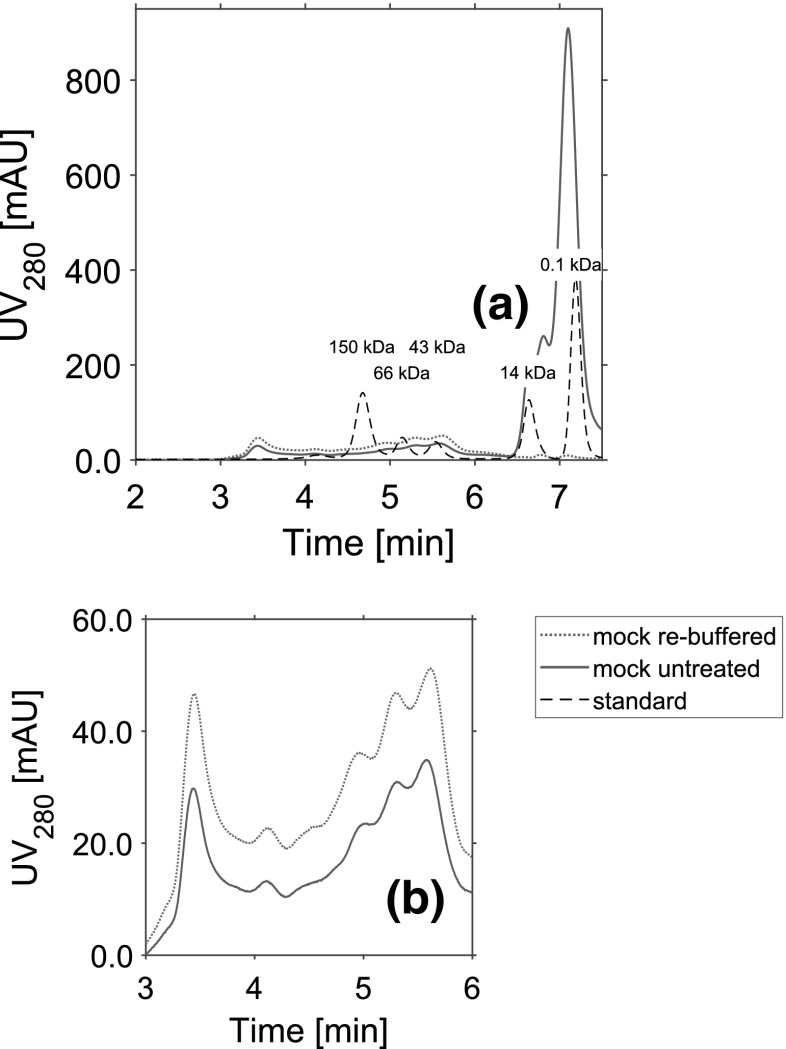
SEC chromatogram of untreated (solid line) and re-buffered (dotted line) mock solution samples. **a** Referred to the size standard (dashed line), molecules above 6.6 min are smaller than 14 kDa. **b** Displays the magnified details of the size distribution of both utilized mAbs

### Pretreatment of the protein stock solution

After filtration, the purified mAb was re-buffered and desalted into the 50 mM tris buffer pH 7.5 using PD 10 desalting columns (GE Healthcare, Little Chalfont, UK). The mock solution was divided into two parts. One part was spiked later to the mAb without further pretreatment. The other part was re-buffered to 50 mM tris buffer pH 7.5 using a KrosFlow Resarch IIi tangential flow filtration (TFF) system (Spectrum Labs, Breda, Netherlands). To restrain larger contaminants like HCP or DNA and deplete small molecules present in the HCCF, the TFF system was equipped with a 10 kDa modified polyethersulfone (mPES) MicroKros$^{\textregistered }$ hollow fiber filter module (Part number: C04-E010-05-S) and an automated back-pressure valve (both Spectrum Labs, Breda, Netherlands). The process was performed with a flow rate of 27 mL/min, a transmembrane pressure (TMP) of 0.6 bar, and a shear rate of 5800 1/s. First, the mock solution was concentrated fivefold in ultrafiltration mode (UF). Subsequently, the mock solution was re-buffered into 50 mM tris buffer over 5 diafiltration volumes (DV). The concentration of both mock stock solutions is listed in Table 1.

**Fig. 5:**
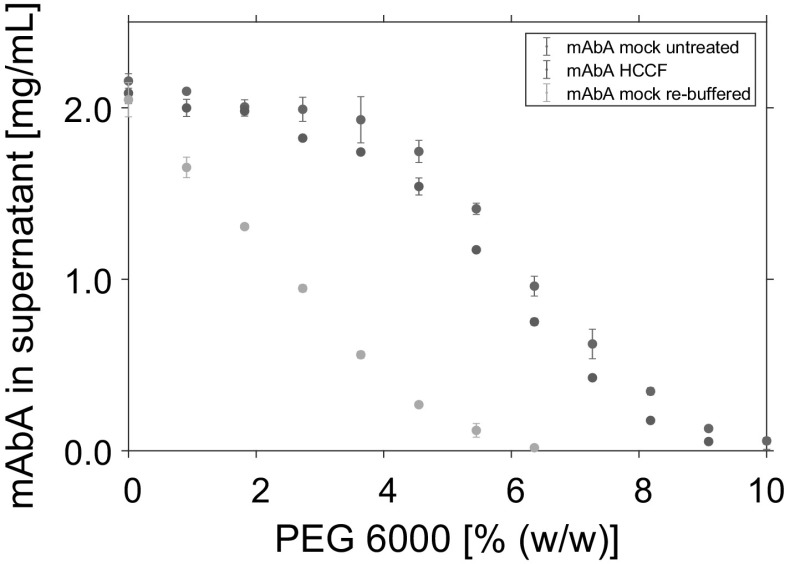
Solubility data of mAbA in the supernatant after phase separation at varying PEG concentrations. Data points represent the mean value of triplicates. Comparison of mAbA provided as HCCF, purified mAbA spiked with untreated mock solution, and purified mAbA spiked with re-buffered mock solution. All experiments were conducted using 50 mM tris buffer at pH 7.5 and a mAbA concentration of 2 mg/mL

### Analytical methods

To determine the component content and size distribution, the UHPLC system ultimate 3000RSLC, controlled with Chromeleon 6.8 (both Thermo Fisher Scientific, Waltham, MA, USA) was used. For mAb concentration and contaminant content, the ultra-high performance chromatography (UHPLC) system was equipped with a Poros analytical Protein A column (Thermo Fisher Scientific, Waltham, MA, USA). The mAb concentrations were calculated by integration of the elution peak area, using calibration curves. The corresponding contaminant content was measured by integration of the flow-through peak. Analytical size exclusion chromatography (SEC) was performed using a TSKgel SuperSW mAb HTP column (TOSOH, Tokio, Japan). For conductivity measurements, a CDM 230 conductivity meter (Radiometer Analytical SAS, Lyon, France) was used. HCP concentration of the mock solutions was determined using a microfluidic CD-based ELISA-like assay on the Gyrolab XPlore station controlled by Gyrolab (Gyros AB, Uppsala, Sweden).

**Fig. 6:**
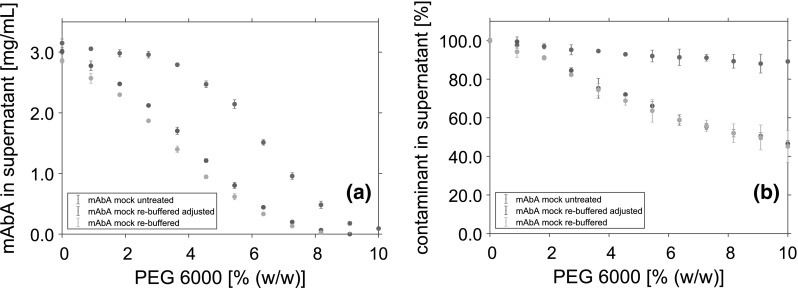
Solubility data of mAbA (**a**) and contaminants (**b**) in the supernatant after phase separation at varying PEG concentrations. Data points represent the mean value of triplicates. All experiments were conducted using 50 mM tris buffer at pH 7.5 and mAbA concentration of 3 mg/mL. The mAbA was spiked with untreated mock solution, re-buffered, and conductivity-adjusted re-buffered mock solution. All samples were adjusted to a HCP concentration of 0.5 mg/mL

### High-throughput method for precipitation screening

Precipitation experiments were carried out on a Tecan Freedom Evo 200 System liquid-handling station (Tecan, Männedorf, Switzerland). The liquid-handling station was equipped with an 8-tip liquid-handling arm, a 96-MultiChannel Arm$^{\textregistered }$ (MCA), a robotic manipulator arm, a Te-Shake orbital shaker, an Infinite$^{\textregistered }$ 200 UV-Vis spectrophotometer (all Tecan, Männedorf, Switzerland), and a Rotanta 46RSC centrifuge (Hettich GmbH & Co. KG, Tuttlingen, Germany). The system was controlled by Evoware 2.5 (Tecan, Männedorf, Switzerland). Excel 2016 (Microsoft, Redmond, WA, USA) was used as data import format and for data storage. All calculations were done using Matlab$^{\textregistered }$ R2018a (The Mathworks, Natick, MA, USA). All experiments were carried out at $22\,^\circ$C, controlled by air conditioning. To avoid evaporation of the systems, the microplates were capped prior to each incubation or centrifugation step. Systems with a total volume of $200\,\upmu$L containing varying mAb, mock, and PEG concentrations were prepared. The PEG concentration was varied in 12 equidistant steps. For mAbA, the PEG concentration was varied from 0 to 10% (w/w), for mAbB from 0 to 14% (w/w). The mAb concentration was varied from 1 to 8 mg/mL in 8 equidistant steps in the case of mAbA, and from 1 to 5 mg/mL in 5 equidistant steps for mAbB. For systems containing mock solution, the mock concentration was adjusted referring to a HCP concentration of 0.5 mg/mL, in each system. When cell culture medium was added to the samples, the added volume was kept equal to that of the mock solution. To avoid systematic errors due to automated pipetting, the position for each system on the 96-well micro plate was randomized. After adding the protein stock solution, the system was incubated for 15 min on the orbital shaker at 1000 rpm to ensure complete mixing of the system. Following that, the system was incubated for additional 15 min incubation time without shaking. To analyze the amount of precipitated protein, the microplate was centrifuged for 30 min at 3400$\times$*g*. Then, the supernatant was sampled and diluted at a ratio of 1:2 or 1:3, depending on the initial mAb concentration to avoid detector saturation. Subsequently, analytics of the generated samples were performed using either UV–Vis spectroscopy for systems containing only mAb, or UHPLC for complex mAb solutions.

**Fig. 7:**
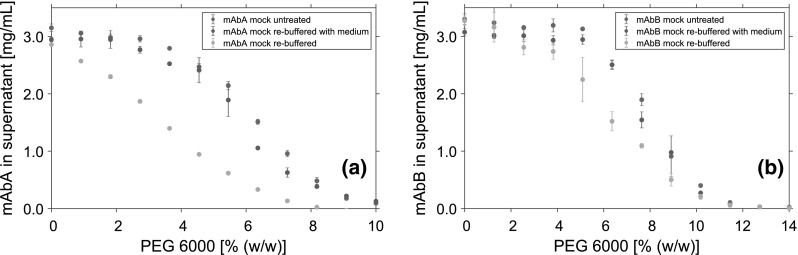
Solubility data of **a** mAbA and **b** mAbB in the supernatant after phase separation at varying PEG concentrations. Data points represent the mean value of triplicates. 3 mg/mL mAb was spiked with untreated mock solution, re-buffered mocksolution, or re-buffered mock solution and cell culture medium

## Results

In this study, the precipitation behaviors of two mAb species using PEG as precipitation agent were investigated. Particularly, the focus was set on the influence of contaminants on the phase behavior of the mAbs and vice versa, as well as on the influence of the mAb on the co-precipitation of the contaminants. In a process based on preparative precipitation, the yield of the precipitation step is defined by the precipitation behavior of the mAb, while the purity is influenced by the precipitation behavior of the contaminants.

To identify variations in the precipitation behavior through the presence of contaminants in mAb solutions and to determine critical process parameters, three sets of experiments were conducted. Precipitation curves of pure mAb solutions were compared to precipitation curves of mAb spiked with untreated and re-buffed mock solution. To imitate a complex mAb feed and, therefore, to investigate the influence of all present contaminants in a cell culture fluid, the mAb was spiked with untreated mock solution. To distinguish between small molecules and molecules larger than 10 kDa, like HCP or DNA, the mAb was spiked with re-buffered mock solution. At the same time, a defined environment could be created, and influencing factors like pH, or buffer components were excluded.

To identify critical process conditions, the influence of conductivity and the cell culture media on the precipitation behavior was investigated in further experiments.

### Solubility of the mAbs

To investigate the precipitation behavior of mAbs, the concentrations of two pure mAb species in supernatant after phase separation were measured. Therefore, the initial mAb and the PEG concentration were varied. The results were compared to the solubility of the same initial mAb concentrations spiked with two different mock solutions (Fig. 2). Solubility data of the two pure mAb species in the supernatant after precipitation at varying initial mAb and PEG concentrations are shown in Fig. 2a, b.

For all investigated mAbA concentrations (Fig. 2a), the mAb was completely soluble at PEG concentrations below 4.5% (w/w). Above this concentration, the solubility was depending on the initial mAb concentrations. Anyhow, all investigated mAbA concentrations approach the same solubility line. At 10% (w/w) PEG, mAbA was completely precipitated for all examined mAb concentrations. Compared to these results, mAbB showed a higher solubility and was completely soluble at PEG concentrations below 7.64% (w/w) (Fig. 2b). Independent of the initial mAb concentration, mAbB was completely precipitated at 14% (w/w) PEG.

To investigate the influence of contaminants on the precipitation behavior, the mAb solutions were spiked with two different mock stock solutions. The added mock solution volume was normalized to a HCP concentration of 0.5 mg/mL. To study the influence of all contaminants present in the cell culture fluid, small ones like sugars, amino acids but also larger ones like HCP or DNA, the mAb solutions were spiked with untreated mock solution. The results are shown in Fig. 2c, d. To distinguish between the influence of an entire cell culture fluid and components with a size larger than 10 kDa, like HCP, or DNA, the mock solution was re-buffered prior to spiking. The results are shown in Fig. 2e, f. Adding contaminants to the mAbs lowered the solubility at each PEG concentration for both investigated mAbs. Thus, the PEG concentration needed for causing precipitation, was lower comparing pure mAb systems with both spiked ones. For systems with re-buffered mock solution, the solubility decrease was even more pronounced. For a concentration of 8 mg/mL mAbA, the initial PEG concentration needed to precipitate the mAb dropped from 4.5% (w/w) PEG for the pure mAb solution to 2.7% (w/w) PEG for the mAb solution spiked with untreated mock solution. For mAbA spiked with re-buffered mock solution, precipitation started at 0.9% (w/w) PEG. For 5 mg/mL mAbB, initial precipitation was observed at 5.09% (w/w) PEG when the solution was spiked with untreated mock solution. Spiking mAbB with re-buffered mock solution led to precipitation at 1.29% (w/w) PEG. Furthermore, there was no approach to the same solubility line detectable for the spiked samples, comparing different initial mAb concentrations. Comparing the precipitation behavior of the same initial mAb concentrations, the spiked samples showed a higher precipitation propensity overall and a flattened solubility line.

Developing a preparative precipitation process, the precipitation propensity of the mAb can be linked directly to the yield of the purification step. For all investigated conditions mAb yield after the precipitation did depend on the initial mAb concentration, as well as on the contaminant composition. For an initial mAbA concentration of 1 mg/mL the highest yield for samples containing pure mAb solution was reached at 10% (w/w) PEG with a value of 86%. However, previous studies have shown that a yield of up to 98% can be achieved after precipitation [34]. In this work, this value was reached for 1 mg/mL mAb concentrations at 10% (w/w) PEG when untreated mock solution was spiked before. For solutions containing re-buffered mock solution 98% yield were reached at 6.36% (w/w) PEG. At an initial mAbA concentration of 8 mg/mL the maximum yield for the pure mAb solution was 97.6%. For solutions containing untreated mock solution the 98% yield was reached at 10% (w/w) PEG. For samples containing re-buffered mock solution 9.08% (w/w) PEG were therefore necessary. For mAbB trends were comparable to that of mAbA. At 1 mg/mL initial concentration of mAbB a maximum yield of 92%, and for 5 mg/mL mAbB a maximum yield of 98% could be observed at 14% (w/w) PEG. For mAbB spiked with untreated mock solution 98% yield was reached at 12.73% (w/w) PEG for all investigated initial mAbB concentrations. For an initial mAbB concentration of 1 mg/mL, spiked with re-buffered mock solution 98% yield was reached at 11.45% (w/w) PEG. At 5 mg/mL initial concentration of mAbB, 12.73% (w/w) PEG was necessary to reach the target yield.

While the precipitation propensity of the mAbs determines the yield, the precipitation behavior of the contaminants affects the purity of the process. Therefore, the solubility of the contaminants was measured as well.

### Solubility of contaminants

The amount of the contaminants in the supernatant after phase separation is shown in Fig. 3. Prior to the addition of PEG, each system was spiked with one of the two utilized mock solutions, normalized to a HCP concentration of 0.5 mg/mL. With increasing PEG concentration, the solubility of the contaminants decreased. This decrease was less pronounced comparing samples spiked with untreated mock solution (Fig. 3c, d) to samples spiked with re-bufferd mock solution (Fig. 3e, f). For systems containing 8 mg/mL mAbA and untreated mock solution, the solubility dropped to 87% of the initial amount at 10% (w/w) PEG. For the same mAbA and PEG concentrations, the amount dropped to 25% when re-buffered mock solution was utilized. For mAbB, a higher contaminant solubility and therefore less precipitation was observed in the same PEG range. Like for mAbA, using re-buffered mock solutions led to a higher amount of precipitated contaminants compared to untreated mock solution.

For samples spiked with untreated mock solution, the solubility showed no dependence on the initial mAb concentration. In contrast, the solubility of the re-buffered contaminants could be connected to the initial mAb concentration in each system. With higher mAb concentrations, lower solubility of the contaminants was detected. This behavior was shown for both investigated mAbs.

In Fig. 3a, b, systems with 2 mg/mL mAbA and mAbB spiked with mock solution are compared to systems containing pure mock solution without addition of mAb. Comparing the samples with mAb and untreated mock solution to pure-mock solution samples, the solubility of the contaminants was lowered by the addition of mAbA. For the pure-mock solution samples, a contaminant solubility of 94.6% of the initial amount was detected at 10% (w/w) PEG. For the samples of mAbA spiked with untreated mock solution, the residual solubility was 88.9%, and for samples containing mAbB, the amount was 94.7%. By comparison, spiking the mAb with re-buffered mock solution lowered the solubility of the contaminants significantly. At 10% (w/w) PEG, 54.0% of the initial contaminant amount was detected for samples containing mAbA, and 49.7% for samples containing mAbB.

Purities after the precipitation step were calculated from the solubility measurements of the impurities and the mAbs. The purity was dependent on the initial mAb concentration, but also on the kind of added mock solution. For the highest investigated PEG concentration and an initial mAbA concentration of 1 mg/mL, a purity of 95% was determined when untreated mock solution was added. At the same initial mAbA concentration, this value dropped to 86%, when re-buffered mock solution was spiked. For an initial concentration of 8 mg/mL mAbA, the purity was 99% for samples containing untreated mock solution compared to 94% for samples containing re-buffered mock solution. Similar trends could also be observed for mAbB. The calculated purities at the highest investigated PEG concentration for samples containing untreated mock solutions were 93% and 99% for an initial mAbB concentration of 1 mg/mL, respectively, 5 mg/mL. For samples containing re-buffered mock solution, the purities were 82%, respectively, 96%, for 1 mg/mL, respectively, 5 mg/mL mAbB.

The observed differences between untreated and re-buffered mock solution suggest that the differences are caused by small molecules depleted through the UF/DF step. To exclude possible influences of the UF/DF step itself or mixing on the liquid-handling station, additional experiments were conducted. Subsequently, the influence of conductivity and fresh cell culture media was examined.

### Comparison of the utilized mock solutions

UF/DF of protein solutions can lead to a higher aggregation level, and the protein size is known to be a significant parameter for PEG precipitation [35]. To prove the integrity of the re-buffered mock solution, the size distributions of both mock solution samples were compared using analytical size exclusion chromatography (SEC), displayed in Fig. 4. Until 6.5 min retention time, both chromatograms show a similar peak profile. After this, the UV signal differs, and for the untreated mock solution, an additional peak was detected. Based on the size standard, molecules eluting after this time have a molecular weight smaller than 14 kDa.

Beside the UF/DF, mixing the solutions on the liquid-handling station is an additional factor in the used experimental setup, which might cause the observed deviations. Spiking mock solution to the mAb solutions comes along with an additional mixing step of mAb and mock solution, comparing the imitated crude feed stock to the actual HCCF. Hence, the phase behavior of spiked mAbA samples was compared to that of mAbA provided as HCCF (Fig. 5). Comparing the precipitation behavior of the HCCF to that of purified mAbA spiked with untreated mock solution, only minor changes could be detected. Compared to these, the systems with purified mAbA and re-buffered mock solution showed a lower solubility for each investigated PEG concentration.

As no influence of the experimental setup could be shown, the different phase behaviors of the mAb and contaminants had to be caused by differences in the composition of untreated and re-buffered mock solution. Due to re-buffering of the mock solution, small molecules were depleted. Consequently, the conductivity of the re-buffered solution decreased from 9.91 to 3.5 mS/cm. To study this effect on the precipitation behavior, the conductivity of the re-buffered mock solution was adjusted to 9.91 mS/cm again, using a 1 M NaCl solution. Afterwards, the solubility of mAbA and the contaminants was measured at a mAbA concentration of 3 mg/mL. The results were compared to the precipitation behavior of the mAb spiked with unadjusted mock solution (Fig. 6). For the mAb solubility (Fig. 6a) as well as for the solubility of the contaminants (Fig. 6b), no significant influence of the conductivity was detected.

By the UF/DF step, not only salts were depleted, but other small molecules like amino acids and vitamins probably as well. Thus, the influence of these small molecules was tested. Therefore, samples containing 3 mg/mL mAb and re-buffered mock solution were spiked with cell culture medium, and afterwards precipitated using PEG (Fig. 7). The used cell culture medium was not identical with the original one used for the fermentation. However, it is composed out of the same group of components: amino acids, vitamins, inorganic salts, and sugars. For mAbA (Fig. 7a), as well as for mAbB (Fig. 7b), adding cell culture medium led to a comparable precipitation behavior of the samples containing re-buffered mock solution and mAb and the samples containing mAb and untreated mock solution.

## Discussion

The effect of contaminants on the precipitation behavior of two different mAbs, an IgG1, here named mAbB, and an IgG2, here named mAbA, was investigated. Therefore, the precipitation curves of the mAbs at varying PEG concentrations were determined. Although mAbA and mAbB are similar in size and pI, a different phase behavior was observed. In comparison, higher PEG concentrations were needed to precipitate mAbB. This indicates that the molecular weight, or the overall surface charge of proteins is not the only factor influencing PEG-induced precipitation. This is consistent with the investigation of Haemmerling et al. [36]. They showed, with the help of quantitative structure activity relationship models (QSAR), that surface characteristics, such as charge distribution, amount of hydrophobic patches, and their distribution on the surface, might play an important role for the precipitation behavior of proteins as well.

The precipitation behavior of the pure mAb solutions was compared to systems which were spiked with one of two different mock solutions as contaminants. To investigate the influence of the complete cell culture medium, the mAbs were spiked with untreated mock solution. The cell culture fluid includes a variety of small molecular weight contaminants which can briefly be divided into four groups: amino acids, vitamins, inorganic salts, and other components, mainly sugars. Side products like fermentation metabolites, or added antifoam, may be included in the cell culture fluid as well. Furthermore, one finds larger contaminants like HCPs or DNA. To investigate the influence of contaminants with a size larger than 10 kDa, the mAbs were spiked with re-buffered mock solution. During the UF/DF step, components with a molecular weight smaller than 10 kDa were removed. The successful removal could be shown with the help of SEC analytic. Additionally, the integrity of the re-buffered mock solution, after the UF/DF step, could be proven. Thereby, changes in the size distribution of the mock solution could be excluded.

To further prove the usability of the developed screening system, the precipitation behavior of mAbA provided as HCCF was compared to systems containing purified mAbA and mock solution. The precipitation behavior of mAbA and the contaminants present in the HCCF could successfully be imitated by mixing purified mAbA with untreated mock solution in a similar proportion. Thus, mixing effects on the liquid-handling station could most likely be excluded.

Focusing on the influence of the contaminants on the precipitation behavior of the mAbs, mAbA, and mAbB showed a similar behavior. In both cases, the addition of contaminants led to a decrease in the solubility of the mAbs. When contaminants were added, precipitation started at lower PEG concentrations. Additionally, the amount of PEG needed for complete precipitation of the antibodies was reduced for both of the mock solutions used. The destabilizing effect could be shown for all investigated mAb concentrations. The higher precipitation propensity was even more pronounced when the mock solution had been re-buffered before. This suggests that, in general, the addition of contaminants destabilizes the antibody, but small molecular contaminants removed through re-buffering the mock solution stabilize the mAb during precipitation. The influence of small molecular contaminants known as osmolytes or co-solutes can be explained with the preferential interaction theory. According to this theory, a preferential binding of co-solutes to the proteins would lead to a stabilization of the system, also known as salting-in [37]. This would mean that the presence of co-solutes either stabilized the mAbs or avoids interaction with larger contaminants present in the re-buffered mock solution. For preparative PEG precipitation, lowering the solubility of the mAb is required. Especially when PEG precipitation is intended to be used as capture step, the amount of required PEG is directly linked to the process costs, as there is no recycling strategy for PEG yet. Therefore, a reduction of the PEG amount is desirable. On the other hand, product loss through incomplete precipitation should be avoided. Changes induced through contaminants are critical factors for process development, in particular when considering a robust process.

While the amount of precipitated mAb can be linked to the yield, the amount of co-precipitated contaminants influences the purity of the product after the precipitation step. In previous studies it was shown, that the purity can be further increased through selective resolubilisation of the product [34]. However, a lower co-precipitation of the contaminants is advantageous, as it reduces the effort for the next step and the risk that the undissolved impurities lead to filter clogging. In this study, investigation of the contaminant solubility showed a slight decrease, comparing mAb spiked with untreated mock solution to pure-mock solution systems. For systems with re-buffered mock solution, the hiring of the precipitation propensity was more pronounced. This is similar to the precipitation behavior of the mAbs. The increase of the precipitation propensity can hence be correlated to the different investigated initial mAb concentrations when re-buffered mock solution was added. An increase in the mAb concentration led to an increase in the precipitated contaminants. This in turn implies a direct or indirect protein–contaminant interaction between the mAbs and the contaminants prior or during the precipitation process.

This leads to a complex problem for process development. Since PEG-induced precipitation of mAbs is mainly used as a capture step, a high yield is of primary significance. For example, for an initial product concentration of 8 mg/mL mAbA, 10% (w/w) PEG was necessary to achieve a yield of 98%. This resulted in a purity of 99% after the precipitation, when untreated mock solution was spiked as contaminant. If re-buffered mock solution was utilized as contaminant, 9% less PEG was required to achieve the same yield. However, this also led to a decrease in the purity of 9.5%. For an initial product concentration of 1 mg/mL mAbA, the effect was different. When re-buffered mock solution was added as contaminant, 36.4% less PEG was required, compared to experiments where untreated mock solution was used, to reach 98% yield. At the same time, the purity was 2% higher. For an initial mAB concentration of 1 mg/mL, 11% more PEG was required to achieve 98% yield when comparing untreated mock to re-buffered mock solution. At the same time, the purity was 21% higher. The examples illustrate how changes in process conditions, such as different product concentrations, changes in the cell culture medium composition, or a change of the product, can affect the purification outcome.

An indication, that protein–contaminant interaction is the reason for the observed differences in yield and purity, is also provided by other studies investigating variations in the HCP level after Protein A chromatography. Sisodiya et al. found that the variation of the HCP level varies between different mAb species and is caused by electrostatic and hydrophobic interactions between the mAb and the HCPs [38]. This confirms the assumption that the presence of large molecular contaminants destabilizes the mAb as well as the contaminant by mAb–contaminant interaction. For systems with untreated mock solution, the correlation between mAb concentration and contaminant precipitation behavior was not possible. Subsequently, modifications during the UF/DF step had to cause the different observed precipitation behaviors.

Two possible explanations for this behavior were investigated. During the UF/DF step, the conductivity was reduced. Beside PEG concentration and pH, the conductivity of the precipitation samples is known to influence the precipitation process [39]. Electrostatic interactions are a conceivable reason for the observed destabilizing effect of added re-buffered mock solution. At the investigated pH of 7.5, both mAbs have a positive net charge while contaminants like HCP, with an average pI of around pH 5.0, are mostly charged negative [40]. Hence, electrostatic attraction might take place and lead to mAb–contaminant interaction and therefore to co-precipitation. However, adjustment of the conductivity did neither show an effect on the phase behavior of mAbA nor on that of the contaminants. This implies either that no electrostatic interaction took place or, more likely, that the higher ionic strength was not the only stabilizing factor.

As changes in the conductivity have not explained the behavior satisfactorily, the other small molecules present in the cell culture fluid, and probably their interplay had to cause the observed variations. By mixing mAb, re-buffered mock solution, and fresh cell culture medium prior to the precipitation, the stabilizing effect of the cell culture media components could be shown. For process development, this is a significant issue, as USP is mostly driven by the needs of higher cell culture, respectively, product titers, and less with respect of the needs for DSP [7]. As side product of USP development, the co-solute composition might change. Furthermore, variations in the HCP profile are reported as a function of the cultivation condition and may be induced during primary clarification [41, 42]. This implies that changes during early production phases are critical for a precipitation process. They can change the precipitation behavior of mAb or contaminants and can, therefore, lead to a variation in yield and purity. Hence, especially for the development of a platform process containing PEG-induced precipitation as the capture step, an integration of up- and downstream steps is of great importance.

## Conclusion

In the present work, the influence of contaminants on the precipitation behavior of mAb and the contaminants during a preparative PEG-induced precipitation step was shown. Higher precipitation propensity could be correlated to the protein–contaminant interaction of the mAb with contaminants larger than 10 kDA. Additionally, a stabilizing effect of the mAb as well as of the contaminants could be linked to small molecules contained in the cell culture media. Other factors like size distribution or conductivity were successfully excluded. It was pointed out that variations in the cell culture media during USP may influence the precipitation performance. This study shows that the amount of PEG, required for a successful precipitation step, can vary by up to 36.2%, depending on the contaminant composition. This theoretically opens up the possibility of reducing the required PEG quantity and thus developing a more economical process. At the same time it helps to estimate the robustness of the process. Also the influence on the purity, after the precipitation step, was found to be a critical factor. Variations of up to 21% were seen for the various process conditions. Therefore, the knowledge gained in this study can probably be used to increase the product purity. It provides the possibility to use co-solutes for manipulation of the precipitation behavior and increase product purity or yield.

The results demonstrate the importance of integrated up- and downstream process development. Deeper knowledge of the phase behavior of complex protein solutions can simplify empirical precipitation process development. Furthermore, understanding the protein precipitation of complex solutions can help to derive new in-silico-based models and therefore, make process development faster and less material-consuming. During the whole purification process, protein–contaminant interactions play a critical role for the product quality. The results obtained in this study may therefore also be important for other unit operations, like chromatographic separations. Hence, further studies should focus on the identification of the stabilizing components in the cell culture media.
